# Influence of Passive Muscle Tension on Electromechanical Delay in Humans

**DOI:** 10.1371/journal.pone.0053159

**Published:** 2013-01-04

**Authors:** Lilian Lacourpaille, François Hug, Antoine Nordez

**Affiliations:** University of Nantes, Laboratory “Motricité, Interactions, Performance” (EA 4334), Nantes, France; University of Zurich, Switzerland

## Abstract

**Background:**

Electromechanical delay is the time lag between onsets of muscle activation and muscle force production and reflects both electro-chemical processes and mechanical processes. The aims of the present study were two-fold: to experimentally determine the slack length of each head of the biceps brachii using elastography and to determine the influence of the length of biceps brachii on electromechanical delay and its electro-chemical/mechanical processes using very high frame rate ultrasound.

**Methods/Results:**

First, 12 participants performed two passive stretches to evaluate the change in passive tension for each head of the biceps brachii. Then, they underwent two electrically evoked contractions from 120 to 20° of elbow flexion (0°: full extension), with the echographic probe maintained over the muscle belly and the myotendinous junction of biceps brachii. The slack length was found to occur at 95.5 ± 6.3° and 95.3 ± 8.2° of the elbow joint angle for the long and short heads of the biceps brachii, respectively. The electromechanical delay was significantly longer at 120° (16.9 ± 3.1 ms; p<0.001), 110° (15.0 ± 3.1 ms; p<0.001) and 100° (12.7 ± 2.5 ms; p = 0.01) of elbow joint angle compared to 90° (11.1 ± 1.7 ms). However, the delay between the onset of electrical stimulation and the onset of both muscle fascicles (3.9 ± 0.2 ms) and myotendinous junction (3.7 ± 0.3 ms) motion was not significantly affected by the joint angle (p>0.95).

**Conclusion:**

In contrast to previous observations on gastrocnemius medialis, the onset of muscle motion and the onset of myotendinous junction motion occurred simultaneously regardless of the length of the biceps brachii. That suggests that the between-muscles differences reported in the literature cannot be explained by different muscle passive tension but instead may be attributable to muscle architectural differences.

## Introduction

Electromechanical delay (EMD) is the time lag between onsets of muscle activation and muscle force production. It reflects both electro-chemical processes (i.e., synaptic transmission, propagation of the action potential, excitation-contraction coupling) as well as mechanical processes (i.e., force transmission along the active and the passive part of the series elastic component, SEC) [1]. The relative contributions of both electro-chemical and mechanical processes involved in EMD has recently been characterized on gastrocnemius medialis [2] and biceps brachii [3], [4] using very high frame rate ultrasound. More precisely, the delay between the muscle electrical stimulation and the onset of muscle fascicles motion has been mainly attributed to electro-chemical processes and the delay between the onset of fascicles motion and the onset of both the myotendinous junction motion and the force production has been attributed to mechanical processes [2], [3], [4]. While the study of gastrocnemius medialis reported a delay of about 2.4 ms between the onset of muscle fascicles motion and the onset of myotendinous junction motion (i.e., the delay to transmit force along the aponeurosis) [2], fascicles and myotendinous junction motion occurred concomitantly in biceps brachii [3], [4]. As proposed by Hug et al. [3], this discrepancy could be explained by architectural differences between these two muscles (pennate vs. fusiform, [5]) and/or different levels of passive tension induced by the experimental setup. Indeed, the ankle joint angle (10° in plantar flexion) used in the study of Nordez et al. [2] induces slight passive muscle tension [6], [7], [8], [9]. In contrast, in the study of Hug et al. [3] the biceps brachii muscle-tendon unit was likely to be slack (elbow joint = 90°) and thus did not produced any passive tension. In this latter case, one would expect a rigid body motion inducing a simultaneous displacement onset of the fascicles and myotendinous junction.

Some studies have reported significant changes in EMD through experimental manipulation of tension in the SEC [6], [10], [11]. More precisely, they showed that EMD is influenced by the time to stretch the SEC when the muscle-tendon unit length is shorter than the estimated slack length (defined here as the length from which the muscle begins to develop passive elastic force). In contrast, these earlier studies also showed that EMD is independent of the passive tension when the muscle-tendon unit is longer than the slack length. One of the main limitations of these studies is that they arbitrarily determined the slack length (i.e., at 90° of elbow angle) [11], or determined it to be at the angle at which no passive joint moment was produced [6]. The utility of this last method is questionable, because the passive joint moment is associated with all structures that cross the joint (i.e., muscles, tendons, skin, articular structures) [12] whereas EMD is only associated with the muscle-tendon unit. Furthermore, none of these studies simultaneously recorded the onset of motion of muscle fascicles and myotendinous junction making them unable to attribute changes to electro-chemical and/or mechanical process. Thus, due to shortcomings in experimental techniques, the relationship between muscle passive tension and the mechanisms involved in EMD has never been investigated.

The aims of the present study were two-fold. First, we experimentally determined the slack length of each head of the biceps brachii using an ultrasound shear wave elastographic technique named supersonic shear imaging (SSI). The main advantage of this technique is that it can be used to accurately estimate passive tension and slack length of an individual muscle [8]. For instance, as biceps brachii is composed of two heads with different proximal insertions one would expect different passive tension within each head at a given joint angle. Secondly, we determined the influence of biceps brachii length on EMD and its mechanisms. We hypothesized that for a muscle-tendon unit length shorter than the measured slack length the onset of fascicle motion would not be different to the onset of myotendinous junction motion (i.e., transmission force as rigid body motion) [3]. For a muscle-tendon unit length longer than the slack length, we hypothesized that a significant delay should exist between the onset of fascicles motion and the onset of myotendinous junction motion, attributed to the time to transmit force along the aponeurosis, as previously shown in gastrocnemius medialis [2].

## Materials and Methods

### Participants

Twelve males volunteered to participate in the present study (age: 21.8 ± 2.3 years, height: 180.5 ± 3.6 cm, body mass: 76.0 ± 6.1 kg). Participants were informed of the purpose of the study and methods used before providing written consent. The experimental design of the study was approved by the Ethical Committee of Nantes Ouest IV and was conducted in accordance with the Declaration of Helsinki (last modified in 2004).

### Instrumentation

#### Ergometer

**Figure 1:**
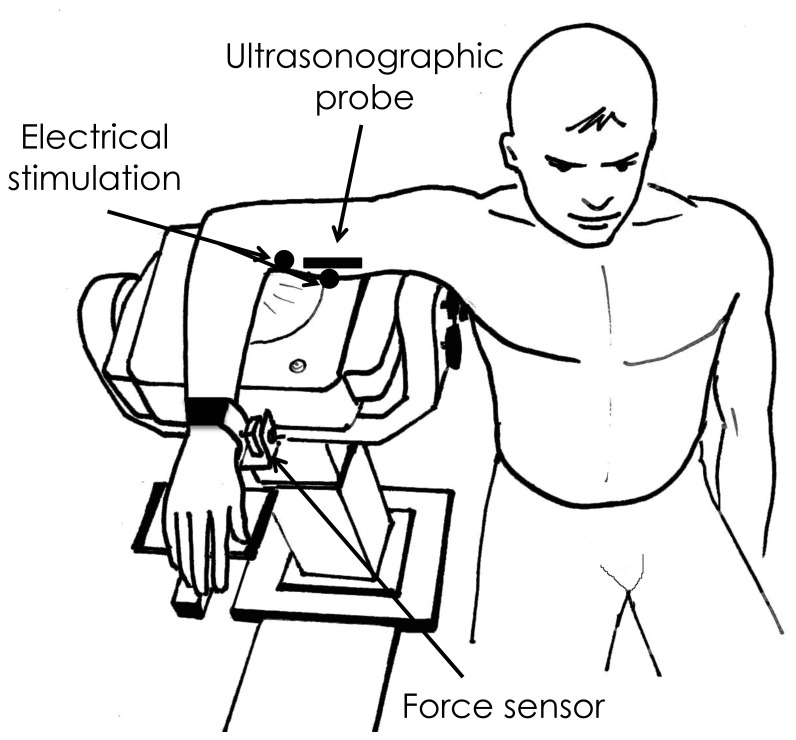
Schematic representation of the experimental setup. Positioning of the subject with shoulder abducted at 90 degrees and forearm placed in a 90 flexed position. The wrist was directly in contact with a force sensor and velcro straps ensured constant contact. Adapted from Lacourpaille et al. (in press) with permission from Elsevier.

Participants sat on an isokinetic dynamometer (Biodex System 3 Research, Biodex Medical, Shirley, USA) with their right shoulder abducted at 90° and with their wrist in a neutral position as described previously [4] (Fig. 1). The torso was strapped to the dynamometer chair to ensure that the participant’s shoulder/trunk position did not change throughout the experiment.

Due to the lack of sensitivity of the isokinetic ergometer to precisely detect the onset of elbow flexion force, a force transducer (SML-50, range: 0–50 Ibf, sensibility: 2 mV/V, Interface, Arizona, USA) was incorporated in the ergometer and connected with Velcro straps to the wrist to ensure constant contact (Fig. 1). Elbow flexion force was digitized at a sampling rate of 5 kHz (MP36, BIOPAC, Goleta, California, USA).

#### Elastography

**Figure 2:**
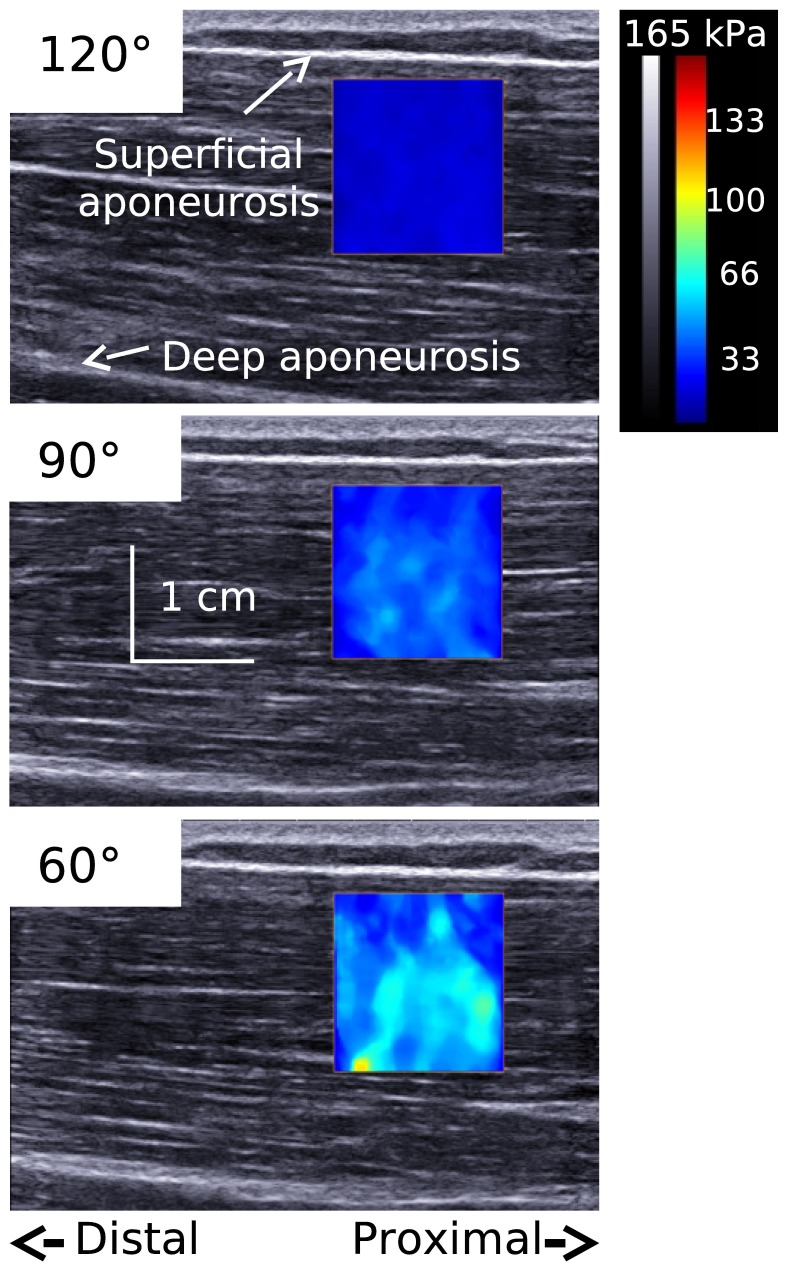
Typical example of shear elastic modulus measurement at different elbow angles. Typical example of shear elastic modulus of the long head of biceps brachii at 120°, 90°, and 20° of elbow joint angle. The colored region represents the map of shear elastic modulus values.

During the first part of the protocol (i.e., passive stretching cycles), an Aixplorer ultrasound scanner (version 4.2; Supersonic Imagine, Aix-en-Provence, France), coupled with a linear transducer array (4–15 MHz, SuperLinear 15-4, Vermon, Tours, France) was used in SSI mode (musculo-skeletal preset) as previously described [13], [14]. Assuming a linear elastic behavior, the muscle shear elastic modulus was calculated as follow:
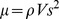
Where ρ is the muscle mass density (1000 kg.m^3^) and Vs is the shear wave speed. As discussed previously [15], [16], the hypothesis of linear material is well accepted in muscle elastographic studies, for both transient elastography [13], [17] and magnetic resonance elastography [18], [19]. Maps of the shear elastic modulus were obtained at 1 Hz with a spatial resolution of 1×1 mm (Fig. 2).

#### Surface EMG activity

Surface EMG electrodes (Delsys DE 2.1, Delsys Inc., Boston, MA, USA; 1 cm interelectrode distance) were placed on the muscle belly biceps brachii and long head of triceps brachii. EMG signals were amplified (ξ1000) and digitized (6–400 Hz bandwidth) at a sampling rate of 1 kHz (Bagnoli 16, Delsys, Inc. Boston, USA). EMG was monitored during the passive stretching cycles, and trials with EMG greater than 1% of maximal voluntary contraction were discarded [9], [20], [21].

#### Electrical stimulation

During the second part of the protocol (i.e., electromechanical delay), elbow flexion was initiated by means of percutaneous electrical stimulation over the biceps brachii. A constant current stimulator (Digitimer DS7A, Digitimer, Letchworth Garden City, UK) delivered a single electrical pulse (pulse duration = 500 µs, 400 V) through two electrodes (2×1.5 cm, Compex, Annecy-le-vieux, France) placed on the main motor point and on the distal portion of biceps brachii [3], [4]. The motor point was determined by detecting the location that induced the strongest muscle twitch with the lowest electrical stimulation intensity. To determine the minimal stimulation intensity required to induce the maximal muscle torque (107 ± 24 mA), the output current was incrementally increased (from 0 mA, with an incremental step of 5 mA) until a maximum torque output was reached.

#### Ultrasonography

To assess the electromechanical delay, a very high frame rate ultrasound scanner (Aixplorer, version 4.2, Supersonic Imagine, Aix en Provence, France) coupled with a linear transducer array (4–15 MHz, SuperLinear 15-4, Vermon, Tours, France) was used in « research » mode to acquire raw radio frequency (RF) signals at 4 kHz. At the start of each ultrasound acquisition, the scanner sent a transistor-transistor logic (TTL) pulse to a train/delay generator (DS7A, Digitimer Ltd, Welwyn Garden City, UK) that generated a TTL pulse to the electrical stimulator with a 48.00-ms delay to have a sufficient baseline to detect the onset of tissue motion. To check the consistency of synchronization throughout the experiments, TTL pulses from both the ultrasound scanner and the train/delay generator were recorded using the same device that recorded the force signal (MP36, Biopac, Goleta, California, USA).

### Protocol

#### Passive tension

To account for a possible effect of conditioning, participants first performed five slow (10°/s) passive loading/unloading cycles between 120° and 20° of elbow flexion (0° represents full extension) that were not analyzed [22]. Immediately after, the ultrasound probe was placed on either head of the biceps brachii (in random order) and participant’s biceps brachii was passively stretched through 2 very slow (2°/s) loading/unloading cycles over the same range of motion. The shear elastic modulus of each head of the biceps brachii was measured during the extension phase. Online EMG feedback was provided to the participants and the examiner. Participants were asked to stay as relaxed as possible throughout the loading/unloading cycles. If EMG activity was observed during the trial, recording ceased, and another trial initiated. However, this did not occur.

#### Electromechanical delay

Immediately after completion of the first part of the protocol, EMD was evaluated at a range of elbow flexion angles. First, five slow (10°/s) passive loading/unloading cycles were performed to account for a possible effect of conditioning (no data were recorded during these cycles). Then, two electrically evoked contractions of biceps brachii (designated as muscle trials and tendon trials, when the ultrasound probe was positioned above the muscle and tendon, respectively) were performed at 11 angles, i.e., in 10° increments from 20° to 120° of elbow flexion, in a randomized order, with one minute of rest between each contraction. Participants were instructed to be fully relaxed prior to each stimulation. During the muscle and tendon trials, the echographic probe was maintained parallel to the muscle fascicles and on the previously localized distal myotendinous junction of the biceps brachii, respectively. Because it was not possible to selectively stimulate one head of biceps brachii by percutaneous electromyostimulation without stimulating the other head, we were not able to determine a specific EMD for each head. Thus, the measured EMD corresponded to the EMD of the whole muscle [3], [4].

### Data Processing

All the data were processed using custom Matlab scripts (The Mathworks, Nathick, USA).

SSI recordings were exported from software (Version 4.2, Supersonic Imagine, Aix en Provence, France) in ‘‘mp4’’ format and sequenced in ‘‘jpeg’’. Image processing was performed to convert the colored map into shear elastic modulus values. The average value of shear elastic modulus over the largest muscular region was calculated for each image. The slack length was visually determined by an experienced examiner for both the short and long head [4].

Ultrasonic raw data (i.e., RF signals) obtained using the very high frame rate ultrasound device were used to create echographic images by applying a conventional beam formation, i.e., applying a time-delay operation to compensate for the travel time differences. These ultrasound images were used to determine the region of interest (ROI; see Fig. 3 of ref [4]) for each contraction, i.e., between the two aponeuroses of the biceps brachii muscle for muscle trials and on the biceps brachii myotendinous junction for tendon trials. Using a one-dimensional cross correlation of windows of consecutive RF signals, the displacements along the ultrasound beam axis (*y*-axis) were calculated [23], [24], [25]. Thus, the tissue motion between the two consecutive images (i.e., particle velocity) was measured with a micrometric precision. Displacements were then averaged over the previously determined ROI, and these averaged signals were used to detect the onset of motion. The onset of tissue motion (for the muscle fascicles and myotendinous junction) and the onset of force production were detected visually [4]. The time (delay) between the electrical stimulation (i.e., beginning of stimulation artefact) and the onset of muscle fascicles motion (Dm), myotendinous junction motion (Dt), and force production (EMD) were calculated for each elbow angle.

**Figure 3:**
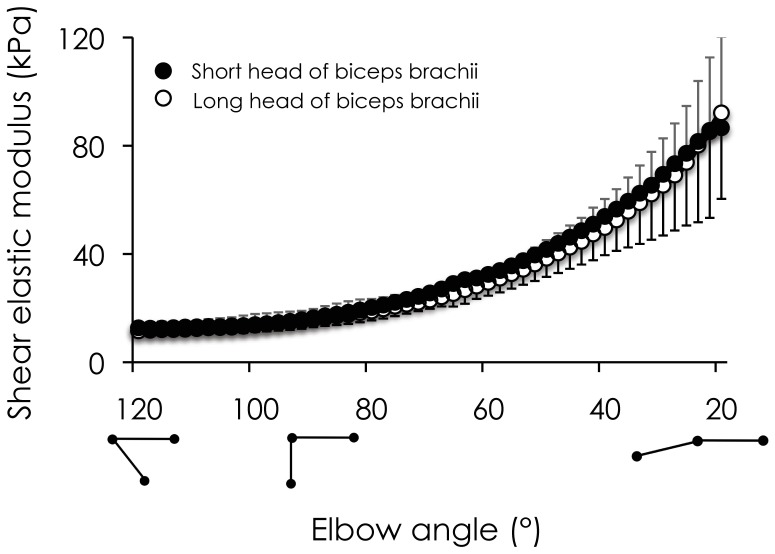
Relationship between shear elastic modulus and elbow joint angle for both heads of biceps brachii. Shear elastic modulus (kPa) was obtained for all participants using supersonic shear imaging during passive loading cycles performed between 20° and 120° of elbow flexion (0° represents full extension). The change of shear elastic modulus for both the short head (mean values in black circles and standard deviation in black lines) and the long head (mean values in open circles and standard deviation in gray) of biceps brachii is represented.

### Statistical Analysis

Normality testing (Kolmogorov-Smirnov) was consistently passed and thus values are reported as mean ± standard deviation. A paired t-test was used to compare the slack length of the two heads of biceps brachii. The effect of probe location [i.e., 4 locations (Dm and EMD for muscle trials and Dt and EMD for tendon trials)] and elbow joint angle [i.e., 11 elbow angles (20, 30, 40, 50, 60, 70, 80, 90, 100, 110, and 120°)] was tested using a two-way repeated measures ANOVA. Post-hoc analyses were performed when appropriated using Scheffe’s method. The statistical significance was set at p<0.05.

## Results

Due to technical problems in the export of high frame rate ultrasound data and amplification of force signals during the experimentation, two participants were not included in the analysis. Results are therefore presented from 10 participants.

### Relationship between Muscle Shear Elastic Modulus and Elbow Joint Angle

Fig. 3 depicts the relationship between shear elastic modulus and elbow joint angle for each head of the biceps brachii. In accordance with previous literature [8], the change in muscles stiffness during the passive stretching was exponential. The smallest elbow flexion angle at which the shear elastic modulus increased (the slack length) was similar between the two heads of the biceps brachii (95.5 ± 6.3° and 95.3 ± 8.2° for the long and short head of the biceps brachii, respectively) (p = 0.99).

### Effect of Elbow Joint Angle on EMD, Dm and Dt

**Figure 4:**
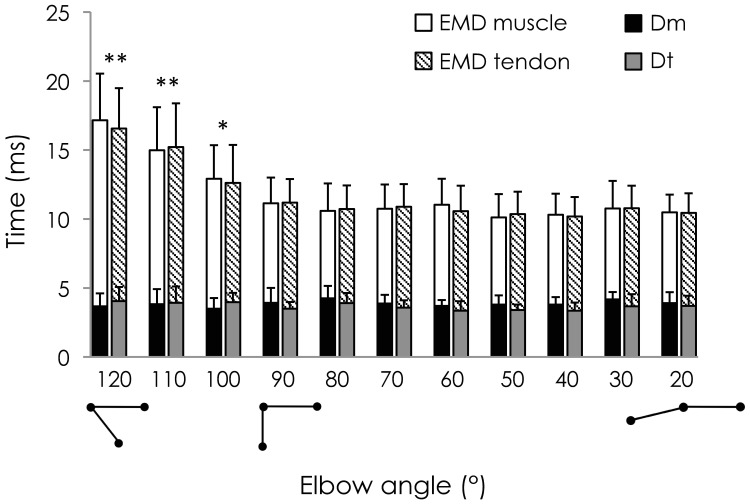
Effect of elbow joint angle on EMD, Dm, and Dt. For each angle, electromechanical delay (EMD), the onset of muscle fascicle motion (Dm) and the onset of myotendinous junction motion (Dt) were determined during an electrically evoked contraction. Two histograms are depicted for each angle (for muscle trials and for tendon trials). EMD for muscle and tendon trials is represented by the addition of filled and open bars (muscle trials) and filled and hatched bars (tendon trials). Dm and Dt are symbolized by the filled bars in black and grey, respectively. *: p<0.05 and **: p<0.001: Significant difference in EMD from the elbow joint angle of 90°.

The influence of the elbow joint angle on EMD and its mechanisms (Dm and Dt) is depicted in Fig. 4. ANOVA revealed a significant main effect of location (p<0.001). More precisely, Dm was significantly shorter than EMD for muscle trials (3.9 ± 0.2 ms vs. 11.8 ± 2.3 ms; p<0.001), and Dt was significantly shorter than EMD for tendon trials (3.7 ± 0.3 ms vs. 11.8 ± 2.2 ms; p<0.001). No significant difference was found between Dm and Dt (p = 0.96) or between EMD measured during muscle trials and tendon trials (p = 0.99).

A main effect of elbow joint angle on the delays was found (p<0.001) corresponding to an overall decrease when the elbow joint was extended. In addition, a significant interaction location x elbow joint angles was found (p<0.001), indicating that Dm, Dt, and EMD were not similarly altered by the elbow joint angle. More precisely, 120°, 110° and 100° of the elbow joint angle induced a significantly longer EMD compared to 90° (i.e., p<0.001, p<0.001, and p = 0.01, respectively). However, there were no significant changes in Dm and Dt across elbow angles (p>0.95 for all the paired comparisons).

## Discussion

The aim of the present work was to determine the slack length of each head of the biceps brachii muscle, and to evaluate the influence of muscle length (relative to this slack length) on the electro-chemical and mechanical processes involved in the electromechanical delay. The results demonstrate that the slack length of both heads of biceps brachii occur at the same elbow angle (≈95°). EMD was significantly longer for the most flexed elbow angles (100°, 110°, and 120°) compared to 90°, and it was not significantly changed for more extended angles, i.e when muscle-tendon length was longer than the slack length. The onset of muscle motion and the onset of myotendinous junction motion occurred simultaneously regardless of the muscle length.

A passive torque-angle curve is classically used to study the behaviour of the muscle-tendon unit *in vivo* [9], [21], [22], [26], [27]. However, this curve is a composite of several structures including agonist and antagonist muscles, tendons, skin, ligaments, joint capsule, etc. [12]. Consequently, it cannot be used to directly estimate the slack length of a given muscle-tendon unit. Maisetti et al. [8] recently showed that the shear elastic modulus of the gastrocnemius medialis can be reliably measured using SSI during the loading phase of passive stretches providing a direct estimation of passive muscle-tendon tension and slack length. Using the same experimental technique, we determined the slack length of the biceps brachii in the present study at about 95°, corresponding to a muscle-tendon length of about 35.1 cm (calculating using the model proposed by Martin et al. [28] and Valour and Pousson, [29]). The main advantage of the elastographic method used in the present study is that it can be easily used to individualize neuromusculoskeletal models. As the muscle-tendon slack length is one of the parameters in Hill-type muscle models [30], [31] this individualization is of great interest. Despite the differences in proximal insertion between the two heads of the biceps brachii and the potential “pre-tension” of the short head of biceps brachii induced by the experimental setup (i.e., shoulder abducted 90°), the slack length of both heads occurred at the same joint angle. Mechanical interactions between muscles have been shown in animals [32], [33] and humans [34]. As the two heads of the biceps brachii are connected by the bicipital aponeurosis and their common distal tendon, it is possible that intermuscular force transmission occurred between the two heads. In other words, passive tension within one head could have been transmitted to the other one, explaining that the slack length occurred at the same angle for each of them.

We report EMD values ranging from 10.2 ± 1.4 ms to 17.2 ± 3.4 ms for 50° and 120° of elbow flexion, respectively. These values are close to those reported in the literature during electrically evoked contractions and confirm that EMD is affected by the muscle-tendon unit length [2], [3], [4], [10], [11], [35]. More precisely, for muscle-tendon unit lengths shorter than the measured slack length (i.e., 95° of elbow flexion), the EMD decreased with increase in elbow joint angle (Fig. 4) until a plateau of 90° of elbow flexion, after which EMD remained stable. As previously suggested by Sasaki et al. [11], the increase in EMD at short muscle lengths (i.e., shorter than the slack length) is likely to be explained by the time required for the muscle to take up the slack within the muscle-tendon unit.

To our knowledge, only Sasaki et al. [11] previously studied the influence of joint angle on electrochemical processes of EMD, which was determined as the delay between the onset of stimulation and the onset of muscle contraction (assessed by accelerometers placed on the skin). They showed that this delay was not influenced by elbow joint angle. However, no study has evaluated the relationship between elbow joint angle and the force transmission between muscle fascicles and myotendinous junction during EMD. The results of the present study demonstrate that Dm and Dt were not significantly altered by the elbow joint angle and occurred concomitantly regardless of the muscle length. Therefore, the discrepancy in the force transmission in EMD between gastrocnemius medialis [2] and biceps brachii [3] previously reported in the literature cannot be due to differences in muscle passive tension induced by the experimental setup. The architectural differences between these two muscles (pennate vs. fusiform) are likely to explain the differences in muscle force transmission suggested by Dm, Dt and EMD measurements.

### Conclusion

This study shows that the slack length determined by SSI does not differ between the two heads of the biceps brachii. Our results also show that the discrepancy in electro-chemical and mechanical processes of EMD between gastrocnemius medialis [2] and biceps brachii [3] is likely due to muscle architecture rather than a difference in passive tension. Both the determination of the slack length by SSI and EMD could be useful to follow changes in mechanical and contractile properties of target muscles particularly affected by neuromuscular disorders or involved in rehabilitation/training programs.
